# Adaptation and Plasticity of Breathing during Behavioral and Cognitive Tasks

**DOI:** 10.1155/2016/2804205

**Published:** 2016-09-29

**Authors:** Nathalie Buonviso, Mathias Dutschmann, Anne-Marie Mouly, Daniel W. Wesson

**Affiliations:** ^1^Centre de Recherche en Neurosciences de Lyon, Codage et Mémoire Olfactive (CMO), CNRS UMR5292, INSERM U1028, Université Lyon 1, 50 avenue Tony Garnier, 69366 Lyon Cedex 07, France; ^2^The Florey Institute of Neuroscience and Mental Health, 30 Royal Parade, Parkville, VIC 3052, Australia; ^3^Department of Neurosciences, Case Western Reserve University, 2109 Adelbert Rd., Cleveland, OH 44106, USA

Since the early work of Pavlov and Anrep [1], it has been apparent that our most basic physiological processes are subject to experience-dependent changes. Additionally, these basic physiological processes, including our biological rhythms of heart rate and breathing, are under the influence of cognitive, sensory, and affective factors. For instance, heart rate orienting responses, the increase in heart beat frequency upon the perception of a novel/arousing stimulus, requires both the detection of the environmental stimulus and the cellular memory which “recognizes” that the stimulus is novel (e.g., [2, 3]). Major questions remain regarding the influence of cognitive, sensory, and affective processes on our biological rhythms and, relatedly, what roles these changes may then entail upon subsequent cognitive and sensory processing.

In this issue, we have gathered groups of investigators sharing common interests in understanding an especially engaging question in neuroscience: the interplay between respiratory rhythm and brain functions. The topics in this special issue span numerous domains which explore the relationship between respiratory rhythm and brain function, including the interplay between respiratory physiology and emotions, the changes in respiration as a function of learning, motor execution, and sensory input, the influence of respiration on brain rhythms, and even early developmental aspects within the respiratory network, to name a few. Understanding these issues is important for understanding a wide variety of fundamental neurobiological questions. Further, alterations in breathing patterns are often observed in instances of neurological disorders in association with cognitive deficits (e.g., [4]). Evidence suggests that the development of remediation tools based on clinical and/or preclinical respiratory function is efficacious [5] and thus understanding more about the interplay between respiration and central brain structures can help inform additional therapeutic strategies. Thus, taken together, these matters highlight the importance of the subject in this special issue.

As a vital function, breathing requires adapting constantly to changing conditions. In this issue, M. Chevalier and colleagues combine electrophysiology and calcium imaging in a brainstem slice preparation to show that mouse embryonic inspiratory pacemakers are already sensitive to neuromodulation and external conditions (i.e., temperature) affecting respiratory network activity.

Brain neuronal network dynamics can be strongly influenced by respiratory dynamics since the periodical nature of breathing can interact with cerebral rhythms (e.g., [6–8]) which may in turn facilitate sensory and/or cognitive processes. In this issue, M. Chatterjee and colleagues use an olfactory bulb slice preparation to test the influence of respiration patterning on bulbar plasticity. They demonstrate that sniff-like electrical stimulation induces LTP/LTD processes at the mitral/granule synapse, possibly endowing the olfactory system with the sensitivity required for fast learning in specific exploration conditions. J. Jessberger and colleagues explore the influence of sleep-wake states in mice upon local field potentials in the main olfactory bulb and how the local field potentials may be entrained with respiration. They present interesting evidence that olfactory bulb local field potentials are entrained to the respiratory rhythm during states of wakefulness and REM sleep, but interestingly not during NREM state sleep.

The structure of the respiratory rhythm during coordinated motor control and during stimulus sampling is highly dynamic and thought to be important for basic physiology as well as shaping sensory input into the brain. Breathing must be coordinated with many other orofacial functions like exploratory sniffing, whisking, licking, chewing, swallowing, and speech/vocalizations. Second, the respiration rate is strongly modulated during sensory exploration, particularly in olfaction. In this issue, G. Coronas-Samano and colleagues perform a remarkably detailed analysis of odor-directed sniffing behavior of mice, aided by video-tracking, in the context of the classic odor cross-habituation test. They report that the more commonly used “video-alone” method of quantifying odor investigation, while being accurate, is not as sensitive and robust as when employing respiratory measures in combination with video. Relatedly, J. A. Alves et al. explored the coupling of respiration and vocalizations in rats during locomotion and bodily movement. It has been well known in other quadrupedal animals that respiration couples with locomotion, something considered adaptive to allow for normal inspiration during trotting and running [9]. Here, J. A. Alves et al. extend this investigation and report that locomotor stride impacts the occurrence of the respiratory rhythm in rats as well. Additionally, M. Deschênes and colleagues synthesize a review on neural circuitry underlying the interplay between sniffing and whisking behaviors. They propose that respiration serves as a “master clock” to couple orofacial sensory input and discuss their recent work exploring neural networks in the ventral medulla which subserves this.

As mentioned earlier, respiration is also impacted by cognition and affective states. In this issue, M. Grassmann and colleagues reviewed the literature on the impact of cognitive load on respiration in human subjects to ask whether respiration can be used as a measure of cognitive load. Their review supports the notion that respiratory rate does not entirely associate with cognitive load. Also, in this issue, M. C. Stoeckel et al. investigate the brain networks involved in the anticipation of dyspnea and find intriguing evidence regarding novel neural substrates underlying the power influence of this affective state on respiration.

In summary, the contributions by the groups who participated in this special issue present novel insights into the complex relationship between respiration and numerous cognitive, affective, and sensory processes. The works together remind us that respiration works bidirectionally with the brain: not only does the brain generate the respiratory rhythm, which shapes the internal neural activity, but also active respiratory control due, for instance, to environmental sensory input can influence subsequent neural activity. Future research into this intricate relationship will provide much needed insights into brain function.

*Nathalie Buonviso* *Nathalie Buonviso* *Mathias Dutschmann* *Mathias Dutschmann* *Anne-Marie Mouly* *Anne-Marie Mouly* *Daniel W. Wesson* *Daniel W. Wesson*
